# Supplementary material for: Creating the funerary landscape of Eastern Sudan
Source: PLoS One. 2021 Jul 7;16(7):e0253511. doi: 10.1371/journal.pone.0253511 (PMC8262793; doi:10.1371/journal.pone.0253511)
Supplement: Attachment — Submitted filename: review_PLOS.pdf [file pone.0253511.s001.pdf]

Review of: PONE-D-21-07238, entitled "Creating the funerary landscape of Eastern Sudan".

Stefano Costanzo and colleagues provide an innovative approach to analyze, visualize, and interpret the archaeological remains of tumuli and tombs in eastern Sudan. The research approach of the authors includes point pattern analysis and the integration of an underlying explanatory covariate using quantitative statistics (R software) and spatial modelling (GIS). The applied methods provide a highly useful tool to model point patterns and to understand the role of environmental explanatory parameters like resource availability, terrain roughness, or other variables. In the paper, the authors aim at tracing the spatial patterns of (visible) burial places as a function of visibility and resource availability/accessibility, which is basically a pragmatic reaction to the distribution of the available raw material in the region. Furthermore, they integrate basic remote sensing techniques, mostly based on satellite imagery to identify and interpret visible archaeological remains.

In general, this article provides a very good overview of the methodical workflow of point pattern analysis and furthermore integrates a new parameter to spatial modelling and statistical methods in landscape archaeology (Neyman-Scott Cluster Process), which allows for the detection of ‘nested clustered’ spatial patterns within larger clusters. That can be considered an interesting and innovative approach to quantitative methods in general and particularly the evaluation of large archaeological datasets. I would also like to particularly emphasize the author’s attempt to not just simply *map and plot* but to include also socio-cultural behavior, perception, and group identity performance into the modelling outlook of their research. Consequently, this paper is an informative contribution to archaeological research in general and I am looking forward to seeing this article published.

However, I would like to discuss some minor points, mostly concerning the structure of the paper (for individual comments, please see below). The paper clearly is a methodical paper, which offers the reader an introduction into current approaches in spatial statistics and modelling techniques (see also the previous research of the authors). For this reason, the actual structure of the paper follows a short introduction into the archaeological and environmental settings, followed by a Material and Methods section and a Workflow and Results paragraph. The introduction would benefit from some reconsiderations about the main goal of the paper. Correct me if I am wrong, but it looks a bit like the archaeological settings are used as canvas to apply in-depth statistical methods. I also have the feeling that some parts of the introduction and some parts of section 2 (mostly archaeology) were written by a different author compared to the rest of the paper (see also stylistic and spelling errors, -> comments below). The environmental section is very well written and provides a sufficient overview of the study area’s properties.

The following section 3 (Material and Methods) provides a short overview of the data acquisition and some outlines of point pattern analysis (PPA). This section is followed by paragraph 4, which describes the workflow and also the results of the analysis. That s a tricky thing and could meet with opposition regarding the structure of the paper. From my individual point of view, I am totally convinced that it is not easy to distinguish the methodical workflow from the results because every single step in the analysis provides new potential results, which then require adjusted methods. The workflow itself can be considered an important part of the results and thus cannot function as a stand-alone section – it would give the impression of a completely detached section and decrease the readability of the paper (or any other paper). On the other side, the authors do integrate a Material and Methods paragraph, which (methodically) renders the results section a mere results section. If the authors can find a smooth way to overcome these structural issues, the overall outlook of the paper would be even more convincing.

General comments (please consider that I am not a native speaker)

Abstract:

41: behavior

46: to decipher

51: maybe introduce cairns as funerary elements earlier to a broader readership of PLOS One

52: which is still embedded. Do you mean today?

1 Introduction:

57: extremely?

60: intricate? Maybe complex or nested?

61: missing reference

62: vegetation cover?

68: usually instead of oftentimes?

67-70: consider rewording this section

73: infrastructure

73: subject to, prone to, vulnerable?

74: as much as more eminent? Like many other?

75: One example is the hilly Kassala region and Eritrean borderland in Eastern Sudan, where…

76: copius scatters? Maybe scattered patterns?

78: that such monuments occur in forms belonging… please consider rewording this phrase

80: missing word. And classified?

Also 80: around the shared characteristic of being round… that sounds odd. Consider rewording.

81: are of instead of have?

82: Another

85: with early authors you mean: Previously, or previous authors, or previous results..

85: please reconsider the wording of ‘tribes’, which has a negative connotation. Maybe ‘groups’ is more elegant or just “Beja people”.

89: , which led to the..

95: what is a traditional GIS approach?

2 Geography and Archaeology

The first part is generally well written and provides a very good overview of the study area!

109: are located, and delete respectively

116: erosive power? What means alternance?

117: extant? You mean the modern? I mean, you are using modern elevation data so I assume modern would be the right word.

120: Archaeological records from the… suggest…

122: particularly

122: Mesolithic versus Mesolithic and Palaeolithic, be concise in the paper.

124: refer to the original references here!

125: what do you mean with relatively sedentary? Reconsider this methodologically.. 'relatively' is not a very precise expression in scientific work..

136: in line 80 you state that they share round characteristics! This is contradictory to what you say here…

140: According to previous results (citation…)

141: they not there

142: Two-storey domed

142-144: maybe use century and not secolo or sec. maybe use 16th century AD -> check the journal guidelines for this.

144-145: Their spatial extent stretches from… to or: They stretch from… to

145: in the north

144-147: this is grammatically wrong and hard to understand. Please rephrase.

151: satellite views is certainly not correct. Imagery or images

3 Material and Methods

This is very well written and summarizes nicely current approaches in spatial analyses and quantitative methods. It shows that the author(s) (maybe the work was split because this paragraph shows differences in linguistic style) are very familiar with the statistic approaches presented in this paper (and in general). For a broader readership and to facilitate the reproducibility of the work, it would be helpful to add some more references to this part.

4 Workflow and results

In this paragraph, the authors are mixing up methodical approaches and subsequent results. I know, it is – particularly when applying a statistical approach – not easy to distinguish into a strictly methodical part and a results/discussion section because the workflow itself can be considered a result per se. In this

case, paragraph 4 is a methodical outline of the paper and represents the core of the research. The paper thus turns into a completely methodical and methodological article – which is a good thing! – but this can also be more explicitly highlighted in the abstract and the introduction.

216: A Kernel Density…

216-219: please add references

242/258/9: igneous vs Igneous and metamorphic. Be concise.

357 add model before hybrid

366: site's ? the site's distribution? Better: site distribution patterns?

369 and others: L-function, K-function instead of L function. Please be concise

379: add *the* before L-function

378-379: why do you think so? Spatial interaction only at small scale? If you just state this without adding a discussion, please cite. However, I am not totally sure that this is actually true…. I know that in quantitative methods, a radius or sigma needs to be determined, which is a totally subjective perspective but there are other many authors applying different radii in their research. This is also dependent on terrain permeability and accessibility and hence needs to be reconsidered – at least with a (small) literature review…

391: its instead of their

437: to reveal. Delete again, delete indeed, exists, resulting, weathering processes. Consider also rewording this whole sentence.. maybe split it in two?

490: again: tribes. Please use groups

Figures:

Fig 1: Consider changing the size and the colors of the archaeological features in the map to increase readability (although I am aware that the manuscript will be published online).

Fig. 3: please add north arrow and a grid
