# Supplementary material for: Creating the funerary landscape of Eastern Sudan
Source: PLoS One. 2021 Jul 7;16(7):e0253511. doi: 10.1371/journal.pone.0253511 (PMC8262793; doi:10.1371/journal.pone.0253511)
Supplement: Attachment — Submitted filename: Response to reviewers_Landscape of Eastern Sudan.docx [file pone.0253511.s002.docx]

*Dear Academic Editor, dear Reviewers and dear Technical Editorial Team,*

*We are very happy about the positive feedback we received for our manuscript, and we thank you all for the time invested and the constructive comments that contributed to its significant amelioration.*

*The manuscript’s readability has improved, and many pieces of relevant literature were added during the revision process.*

*In the following you will find our replies to comments and suggestions from all parts.*

-The Technical Editorial Team asks to amend the Acknowledgments section and the Funding Statement in accordance with PLOS guidelines

The revised manuscript was amended accordingly.

-The Technical Editorial Team states “We note that Figures 1, 4, 7 and 9 in your submission contain map/satellite images which may be copyrighted.”, asking for a written permission by the copyright holder.

Figure 1A and 1D contain Digital Elevation Data that is freely available at German Aerospace Center (DLR) (2018): TanDEM-X - Digital Elevation Model (DEM) - Global, 90m. [https://doi.org/10.15489/ju28hc7pui09](https://www.google.com/url?q=https://doi.org/10.15489/ju28hc7pui09&sa=D&source=editors&ust=1620402089601000&usg=AOvVaw1SxrEAJD7vsq5R3JZzjiUD).

Figure 1B is a modified extract from fully Open Access content previously published by Stefano Costanzo <https://doi.org/10.1080/17445647.2020.1869112>.

Figure 1C contains Landsat-8 satellite images freely available at <https://www.usgs.gov/>.

Figure 4 does not contain maps, it is a dataset-derived density estimation. Original content created by Filippo Brandolini with Rstudio.

Figure 7 does not contain maps, they are graphic renditions of Digital Elevation Model-derived variables employed in the analyses. Original content created by Filippo Brandolini with GRASS GIS and Rstudio.

Figure 9 does not contain maps, it is a dataset-derived fitness estimation. Original content created by Filippo Brandolini with Rstudio.

Dr. Michael Kempf suggested several language and sentence structure improvements, which we gratefully accepted - apart from very few cases discussed below. We thank him, as none of us is a native speaker!

-He states that we submitted a methodical paper, observing that during paragraph 4 (Workflow and results) <<the authors are mixing up methodical approaches and subsequent results>> and that <<The paper thus turns into a completely methodical and methodological article>>, although acknowledging the difficulty in separating workflow and results - because the workflow is a result per se when applying this kind of step-by-step geospatial analysis. He thus suggests finding a smooth way to overcome this issue.

While we understand that the article’s structure is slightly anomalous, we don’t agree on it being a completely methodological one, but rather a methodological-applicative case study whose incipit was given by the vast archaeological dataset – not the other way around. This led to new discoveries on two sides: from one hand, we investigated some aspects of the largely unknown funerary ethnoarchaeology of Eastern Sudan, while on the other hand we discovered the applicability of a previously untested statistical tool (the NSC) to humanities. With this premise, we think that our *Workflow and results* paragraph is a suitable depiction of the process that led us from the preliminary evaluation of our raw data to the choice of the NSC process for the modelling of the qubbas’ spatial pattern, which came upon trial and error and exhaustive theoretical preparation. During the initial writing of the article we tried to follow the classic *Methods-Results-Discussion-Conclusions* layout with a sharp separation between methods (steps) and results, which however spoiled the readability of the paper for any archaeologist who’s not familiar with R and advanced statistics, requiring an inevitable back and forth between paragraphs to keep track of the process.

For these reasons, we prefer not to change the structure of Paragraph 4 – *Workflow and results* within the manuscript. However, we tried to overcome the issue by adding a brief foreword about the benefits of Paragraph 4’s structure in the last section of *Introduction* (largely reworded following Rev. 2’s suggestions), stressing the concept that it is designed as a supporting narrative to the deposited R script *(“…a workflow that is presented in paragraph 4 as an easily replicable step-by-step procedure*”). Indications to the incremental steps of Paragraph 4 were added within the text of Paragraph *3.2 – Point Pattern Analysis* in the form of upcoming paragraph numbers (lines 196, 197 and 205).

Moreover, we would like to add that a similar approach was used by Knitter, Nakoinz, Carrero-Pazos etc. in the relevant literature that we cite in Paragraph *3.2 – Point Pattern Analysis.*

-General comments

***ABSTRACT:***

-41: behavior

Changed in the revised manuscript

-46: to decipher

Changed in the revised manuscript

-51: maybe introduce cairns as funerary elements earlier to a broader readership of PLOS One

Since we don’t use it anywhere else in the manuscript, we substituted the term “cairns” with “monuments”

-52: which is still embedded. Do you mean today?

Yes, we meant today. We reworded the sentence to prevent misunderstandings.

***1 INTRODUCTION:***

-57: extremely?

Removed from the revised manuscript

-60: intricate? Maybe complex or nested?

We substituted ‘intricate’ with ‘complex’ in the revised manuscript

-61: missing reference

Reference added

-62: vegetation cover?

Changed in the revised manuscript

-68: usually instead of oftentimes?

Changed in the revised manuscript, the section was reworded

-67-70: consider rewording this section

The section was reworded from 65 to 71, adding bibliographic suggestions provided by Prof. Julia Budka (Rev. 2)

-73: infrastructure

Changed in the revised manuscript

-73: subject to, prone to, vulnerable?

‘Prone to’ was used in the manuscript

-74: as much as more eminent? Like many other?

This was a subtle provocation towards the tendency of famous (to the general public) sites to overshadow lesser known ones when public awareness about conflict- and land mismanagement-driven destruction is risen. The sentence was reworded for smoother reading.

-75: One example is the hilly Kassala region and Eritrean borderland in Eastern Sudan, where…

Changed in the revised manuscript

-76: copius scatters? Maybe scattered patterns?

‘Copious scatters’ was substituted with ‘thousands’ in the manuscript

-78: that such monuments occur in forms belonging… please consider rewording this phrase

The sentence was reworded

-80: missing word. And classified?

The sentence containing this typo was reworded

-Also 80: around the shared characteristic of being round… that sounds odd. Consider rewording.

The sentence was reworded taking into consideration Dr. Kempf’s observation to line 136. The term ‘round’ was used improperly instead of circular, to describe the shape of the base of the tumuli. ‘Ring’, ‘disk’, ‘cone’, ‘heap’, as newly stated in the reworded sentence at line 136, refer to the raised portion of tumuli.

-81: are of instead of have?

Changed in the revised manuscript

-82: Another

Changed in the revised manuscript

-85: with early authors you mean: Previously, or previous authors, or previous results..

‘Initial investigations’ was used in the manuscript instead of ‘Early authors’, because the works we cite were published in 1922 and 1952 and were the first ones on the topic

-85: please reconsider the wording of ‘tribes’, which has a negative connotation. Maybe ‘groups’ is more elegant or just “Beja people”.

Noted and changed to ‘groups’ in the revised manuscript, we are grateful to the reviewer for pointing this out as it could have sparked unintended outrage.

-89: , which led to the..

Changed in the revised manuscript

-95: what is a traditional GIS approach?

’Traditional’ removed from the manuscript as it was redundant and didn’t really have a meaning within the sentence.

***2 GEOGRAPHY AND ARCHAEOLOGY***

-109: are located, and delete respectively

Changed in the revised manuscript

-116: erosive power? What means alternance?

’Erosive power’ added to the manuscript instead of ‘erosive action’. ‘Alternance’ was wrong and changed with ‘alternation’, meaning a sawtooth-like alternation of shallow valleys and ridges.

-117: extant? You mean the modern? I mean, you are using modern elevation data so I assume modern would be the right word.

‘Extant’ is a commonly used term in geomorphology, where it refers to palimpsests of geological/physiographic/hydrographic features resulted from past and current environmental processes that are readable in the present-day – extant – landscape.

-120: Archaeological records from the… suggest…

Changed in the revised manuscript

-122: particularly

Changed in the revised manuscript

-122: Mesolithic versus Mesolithic and Palaeolithic, be concise in the paper.

’Mesolithic’ was substituted with ‘prehistoric’ at line 122

-124: refer to the original references here!

We thank Dr. Kempf for pointing out that the original references may be of great interest for the reader, but because (Manzo, 2017) is a seminal monography that is mostly based on yearly reports and sites gazetteers from the past 40 years we prefer not to include too much minor bibliography. The most relevant references contained therein were already included in our manuscript [23-31].

-125: what do you mean with relatively sedentary? Reconsider this methodologically.. ‘relatively’ is not a very precise expression in scientific work..

Dr. Kempf’s observation is correct. ‘Relatively’ was removed from the manuscript, and the statement was slightly reworded with the addition of a supporting reference regarding prehistoric sorghum domestication in the region.

-136: in line 80 you state that they share round characteristics! This is contradictory to what you say here…

The sentence was reworded to match with line 80.

-140: According to previous results (citation…)

Suggestion accepted but we preferred to say ‘According to previous observations’, which seems more appropriate for the content of those publications, which were in fact brief notes on field surveys.

-141: they not there

Changed in the revised manuscript

-142: Two-storey domed

Changed in the revised manuscript

-142-144: maybe use century and not secolo or sec. maybe use 16th century AD -> check the journal guidelines for this.

Changed in the revised manuscript accordingly

-144-145: Their spatial extent stretches from… to or: They stretch from… to

See ‘144-147’ below

-145: in the north

See ‘144-147’ below

-144-147: this is grammatically wrong and hard to understand. Please rephrase.

The sentence was rephrased and simplified. In the revised version we indicated the mentioned locations with geographic coordinates, instead of distances and directions from Jebel Maman.

-151: satellite views is certainly not correct. Imagery or images

Changed in the revised manuscript


***3 MATERIAL AND METHODS***

Following Dr. Kempf suggestion, we added few more references along the paragraph 3 “*Material and Metods*”. In particular, the fundamental work of Carrero Pazos et al. 2019 (see *References*).

***4 WORKFLOW AND RESULTS***

-216: A Kernel Density…

Changed in the revised manuscript

-216-219: please add references

Added in the revised manuscript

-242/258/9: igneous vs Igneous and metamorphic. Be concise.

We are not sure about this observation; is Dr. Kempf asking to avoid using capital and lowercase letters for the same terms is various parts of the manuscript? If this is the case, we uniformed to lowercase.

-357 add model before hybrid

Changed in the revised manuscript

-366: site’s ? the site’s distribution? Better: site distribution patterns?

’Spatial patterning of sites’ distribution’ was changed with ‘sites’ distribution pattern’

-369 and others: L-function, K-function instead of L function. Please be concise

Changed in the revised manuscript

-379: add the before L-function

This sentence was removed. Please refer to ‘378-379’ for further explanation

-378-379: why do you think so? Spatial interaction only at small scale? If you just state this without adding a discussion, please cite. However, I am not totally sure that this is actually true…. I know that in quantitative methods, a radius or sigma needs to be determined, which is a totally subjective perspective but there are other many authors applying different radii in their research. This is also dependent on terrain permeability and accessibility and hence needs to be reconsidered – at least with a (small) literature review…

Limiting the radius max to 1.5 Km was merely a “strategic” choice for the plot visualization and to reduce the computing time of the process. However, we agree that this part could be misinterpreted by readers, so we decided to remove it completely and to calculate L and cross G functions without any radius limit. We updated the manuscript with the new images (revised Fig. 8 and 10), and we amended the R script code as well. The results are substantially the same, but more thoroughly represented! We are grateful to Dr. Kempf for this suggestion.

-391: its instead of their

Changed in the revised manuscript

-437: to reveal. Delete again, delete indeed, exists, resulting, weathering processes. Consider also rewording this whole sentence.. maybe split it in two?

The sentence was simplified and reworded as suggested by Dr. Kempf

-490: again: tribes. Please use groups

Changed in the revised manuscript


***FIGURES:***

-Fig 1: Consider changing the size and the colors of the archaeological features in the map to increase readability (although I am aware that the manuscript will be published online).

We agree with this suggestion: the Landsat base satellite image has been slightly desaturated, and the coloured symbols of the archaeological dataset made brighter. This should increase the readability without having to zoom into the picture.

-Fig. 3: please add north arrow and a grid

This Figure already has north arrow and a scale! They are a bit small, but they are clearly visible in the 300dpi .tiff picture we uploaded…maybe the reviewer is looking at the scaled-down pictures used to build the pdf for the review.

*On our own initiative, we lightly revised Fig. 4 (“(ppl)” was deleted from the headings) and Fig.6 (the layout of the histograms was changed to improve readability).*

*Moreover, all the originally submitted images were processed through the* Preflight Analysis and Conversion Engine (PACE) *for optimization, as recommended by PLOS.*

Prof. Julia Budka asks to elaborate on, and maybe reconsider, a few key points of our research and word choices. Moreover, she provided precious insights and literature to include in our manuscript in order to reference to other regions in Sudan, where our proposed research method may be applicable due to comparatively similar amounts of archaeological evidence or remoteness – thus needing partly desk-based research.

-Line 94 and passim: Could you elaborate why „monumental funerary landscape of Eastern Sudan“ is an appropriate term? Why and how are these tombs monumental? What is your reference point here, thinking of other monumental stone monuments etc.

When we say ‘monumental funerary landscape of Eastern Sudan’ we do not mean “a landscape containing large monuments”, but rather the impressive landscape created by the sheer amount of tumuli and qubbas, as much as the striking effect that such landscape has on the traveller when observed within the scenery of the region. While it is true that neither the tumuli nor the qubbas, taken individually, are monumental - speaking of size, ornaments etc -, when observed as a clustered unity of thousands of elements spreading across several kilometres at the foothill of the rocky mountains of the Red Sea Hills they can truly be considered a monumental manifestation.

In an effort to avoid cluttering, this explanation was achieved in the revised manuscript by rearranging and partially rewording the last section of the *Introduction* paragraph, with the addition of some recent literature on stone structures/burials/megaliths in the MENA regions.

-Line 100: Maybe you want to refer for the „formation of funerary landscaped in arid regions“ to recent work in the Fourth Cataract or in the Bayuda? See below

All references recommended by Prof. Budka have been included in the revised manuscript within various sections of the *Introduction*.

-Line 416: Since you employed the NSC models, I completely understand that it was tempting to use „galaxy-like clusters“ for your tomb settings – but could you maybe explain what „galaxy-like“ really means here? The same holds true for line 511 “cosmogony” – as much as I like this, is it really an appropriate term for the burialscape of Eastern Sudan? If so, why?

We chose this self-explanatory comparison because the NSC tool was originally designed for the field of cosmology, but Prof. Budka’s observation prompted us to add an explanation at line 416 to prevent misleading for the future reader. We believe that ‘galaxy-like clusters’ is appropriate in our case-study because, as freshly explained in the revised manuscript, hundreds of *offspring* tombs were found to gravitate around very few undetectable (invisible) *parents*, in turn brought together by the constraints of the natural settings - like stars revolve in galaxies that form higher hierarchical gravitational systems. With this new addition, and another brief recall to the concept at line 517, the expression ’Cosmogony of burials’ as part of the *Conclusions* Paragraph title in our opinion becomes fully legitimate without the risk of being misinterpreted as an astroarchaeology attempt.

-Line 417: Could you elaborate what the precise socio-cultural choices were? In what respect are they “precise”? See also your conclusion where a number of questions are still left open and will only be answered by fieldwork.

Prof. Budka’s question opens to interesting considerations, but honestly we used the term ‘precise’ improperly, not realizing it was an Italian-English calque… Lacking a thorough ethnoarchaeological assessment (as stated at line 494 original manuscript) we cannot speculate on the topic, and given the general meaning of the sentence where the term ‘precise’ was included we opted to just remove it to make the paragraph match with the open questions of our conclusions.

-Line 498ff: Are there any means to explain the comparably very low number of tumuli compared to qubbas? How those this difference in quantities affect or not affect your results and proposed narrative of a continuous use/occupation?

In general, the lower number of tumuli, compared to qubbas, may reside in a hypothetically lower population density at the time of their realization, but the lack of information on this topic (line 503 original manuscript) allows no speculation. Nevertheless, we think that it does not affect our discussion and conclusions because, despite their lower number, the tumuli are evenly distributed in our selected area, which is geographically unitary throughout. Moreover, we would like to add that, while remotely compiling the dataset, the possible erosion/destruction of tumuli (as well as qubbas) was of course taken into consideration. We tackled the issue by comparing multiple hi-res satellite images with field-observed badly eroded monuments, achieving the successful identification of elements comprising all grades of conservation. When we compare our computed results with the available historical (*Dahl & Hjort-af-Ornas 2006)* and ethnographic (*Krzywinski 2012)* works – all cited in the manuscript – we can reasonably consider our narrative of continuous use convincing despite the lower number of tumuli, as no explicit occupation gap appears to have taken place in the region for the past 2000 years.
